# The impact of corporate governance on the total factor productivity of pharmaceutical enterprises: a study based on the fsQCA method

**DOI:** 10.1038/s41598-024-52751-8

**Published:** 2024-02-08

**Authors:** Liquan Gao, Fei Dong

**Affiliations:** 1 First Affiliated Hospital, Heilongjiang University of Chinese Medicine, Harbin, 150040 China; 2 The 2nd Affiliated Hospital of Harbin Medical University, Harbin, 150001 China

**Keywords:** Environmental economics, Socioeconomic scenarios

## Abstract

The pharmaceutical industry is an important industry for the national economy and the people's livelihood, which is not only beneficial to the people's livelihood, but also has huge commercial value. How to promote the development of Chinese pharmaceutical industry is an urgent problem to be solved. In this study, 47 listed pharmaceutical companies are taken as cases, and Qualitative Comparative Analysis of Fuzzy Sets (fsQCA) is used to analyze the influence of five antecedent conditions on the total factor productivity of pharmaceutical enterprises from the perspective of corporate governance, and to explore the composition to Total Factor Productivity (TFP) improvement. The results are as follows. First, single corporate governance factor does not constitute the necessary condition to improve the TFP of pharmaceutical enterprises. Second, there are three configurations of high TFP of pharmaceutical enterprises, among these, two configurations belong to regulatory constraints type and one configuration belongs to the active board type. There is only one configurations to low TFP of pharmaceutical enterprises: the passive board. Based on the perspective of configuration, this paper discusses how corporate governance drives TFP improvement in pharmaceutical enterprises, which can provide systematic thinking and practical guidance for each company to promote its TFP improvement according to its own corporate structure.

## Introduction

**Figure 1:**
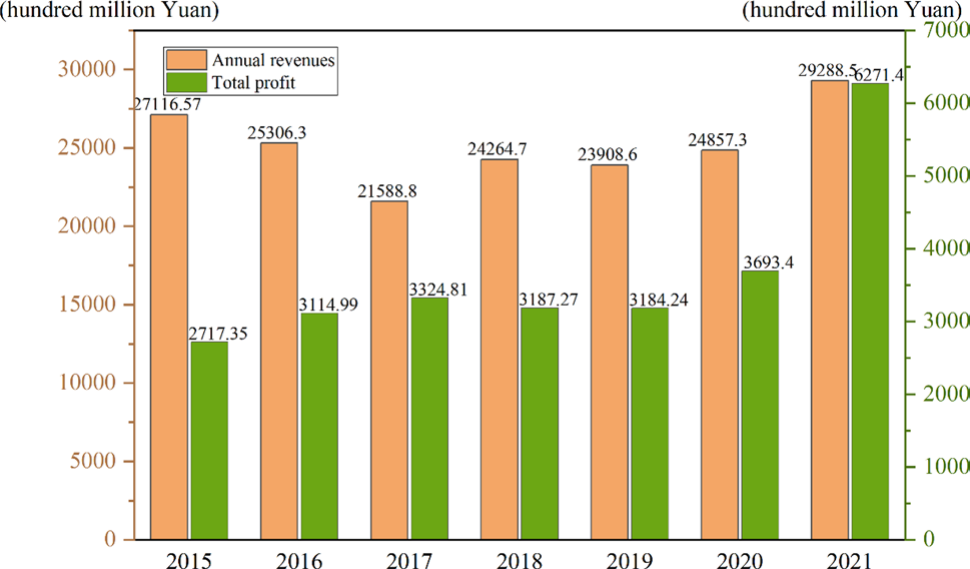
Total revenue and profit of China's pharmaceutical industry from 2015 to 2021.

Drugs are directly related to people's well-being. With the improvement of people's living quality and the serious social problem of population aging, the pharmaceutical industry has gained greater development and more attention^1,2^. China's pharmaceutical industry is growing slowly, and data from the National Bureau of Statistics (NBS) shows that from 2015 to 2021, the operating revenues of Chinese pharmaceutical companies show a "U" shape, which can be divided into three phases: a recessionary period from 2015 to 2017, a slow-growth period from 2017 to 2020, and a fast-growth period from 2021. In the first period, pharmaceutical companies may have poor operating income due to rising energy and raw material prices and increased competition in payment structures. In the second period, pharma companies continue to have poor growth. In the third period, pharmaceutical companies begin to grow rapidly. Figure 1 shows that the compound growth rate of operating income and the compound growth rate of total profit were 1.29% and 15.00% respectively from 2015 to 2020. Total profit of 627.14 billion yuan was realized in 2021, a year-on-year increase of 69.8%. Overall, the development of China’s pharmaceutical industry is relatively slow and the sustainability of future development is unknown. However, it is worth noting that the growth rate of China's pharmaceutical industry is gradually decreasing^3^, and the overall market share of Chinese pharmaceutical enterprises is very low, both in the Chinese market and in the global market. This means that the innovation ability and total factor productivity (TFP) of Chinese pharmaceutical enterprises may be relatively low. This phenomenon is caused by many factors.

Firstly, affected by policy changes, rising energy and raw material prices, and intensifying competition, Chinese pharmaceutical companies face more uncertainties in cost and profitability while expanding their total volume, which seriously dampens their enthusiasm for research and development (R&D)^2,4^. Government policy adjustments may lead to changes in the R&D direction of pharmaceutical companies, and may even restrict or cancel certain R&D projects, which exposes companies to more risks and uncertainties in their R&D decisions^5^. As competition in the market intensifies, firms may focus more on investment in areas such as marketing and sales in order to remain competitive in the competition, while investment in research and development (R&D) may decrease accordingly^6^. Secondly, most of Chinese pharmaceutical manufacturing enterprises are production-oriented enterprises and many drugs they produce are generic drugs, which cannot obtain the monopoly income. The low level of enterprises’ R&D leads to low production efficiency^7^. The fact that most of the drugs are generics means that companies are unable to enjoy the exclusive market gains from patents. The generic drug market is highly competitive and price competition is intense, which puts a lot of pressure on the profitability of companies^8^. Thirdly, the governance structure of the company may have some drawbacks. The unreasonable corporate governance will lead to the loss of competitiveness and vitality of enterprises^9^, and it will seriously hinder the sustainable development of China's pharmaceutical industry in the long run. An irrational corporate governance structure may lead to a lack of efficiency and transparency in the decision-making process. Over-centralization of power or poor information flow may impede the implementation of efficient decision-making and slow down an enterprise's ability to respond to changes in the market^10^. Therefore, combined with the actual development of pharmaceutical enterprises, it is of great research value to explore the configurations of improving TFP and promote the sustainable development of pharmaceutical enterprises.

At present, the main methods for measuring TFP are Stochastic Frontier Analysis (SFA) and Data Envelopment Analysis (DEA). Among them, data envelopment analysis does not need to determine the weights in advance when calculating efficiency assessment, which avoids the intervention of subjective factors and reduces the uncertainty in the evaluation process. So it is more suitable for measuring the TFP of pharmaceutical enterprises. The existing literature shows that there are more studies exploring the relationship between corporate governance and TFP, but there is a lack of studies for pharmaceutical enterprises. The research objective of this paper is to explore a path that helps to improve TFP of pharmaceutical enterprises by revealing the relationship between corporate governance and TFP of pharmaceutical enterprises, so as to provide effective corporate governance recommendations for pharmaceutical enterprises, which will help them to improve competitiveness and long-term sustainability. As the internal factors of enterprises are controllable, this paper takes 47 listed Chinese pharmaceutical enterprises as cases to examine their TFP, adopting Qualitative Comparative Analysis of Fuzzy Sets (fsQCA). This paper explores reasonable configurations to promote the TFP of pharmaceutical enterprises from the perspective of corporate governance structure for the first time. This paper can provide some reasonable and effective suggestions for the further development of Chinese pharmaceutical enterprises.

The rest of the structure of this paper is mainly the following parts: The second part is the literature review and research hypothesis. This part summarizes the literature related to the research topic and proposed the hypothesis. The third section describes the study design and summarizes the data. The fourth part is the measurement of TFP in pharmaceutical enterprises, the fifth part is the research methods and data, the sixth part is the empirical analysis, and the last part is the conclusion and policy recommendations.

## Literature review

### Research on TFP of pharmaceutical enterprises

In academia, most scholars use various deterministic frontier models and stochastic production frontier models for TFP, which is used to evaluate the efficiency of decision-making units in converting inputs into outputs. Stochastic front methods are parametric in nature, while deterministic front methods can be nonparametric. The two methods are Stochastic Frontier Analysis (SFA) and Data Envelopment Analysis (DEA) respectively. In this way, DEA method and SFA method are mainly used to study the efficiency of pharmaceutical industry in academia. A study using the SFA model to measure TFP. Gao Jiangang (2014) analyzed the R&D elasticity and technical efficiency of listed pharmaceutical and biological companies in Shanghai and Shenzhen by using SFA method. The results showed that each 1% increase in R&D inventory expenditure can increase the output value of the industry by 0.11% to 0.17%. This conclusion is basically consistent with the relevant research conclusions at home and abroad. The operating efficiency of the pharmaceutical and biological industry is still relatively low, and there is large room for improvement.

However, due to the nature of the pharmaceutical industry data and research, it is difficult to assume a clear functional form for basic production techniques. That is to say, it is difficult to meet the basic prerequisites of parametric methods. Therefore, more scholars tend to use DEA to measure the efficiency of the pharmaceutical industry. DEA is a non-parametric method and does not require any functional form assumptions^11^. Moreover, DEA models are still evolving, and there are now two-stage DEA models, dynamic DEA, and DEA-ML. Sun (2011) used DEA method to analyze the efficiency and productivity growth of six high-tech industries (including biotechnology industry) in Hsinchu Industrial Park, Taiwan, China from 2000 to 2006, and pointed out that industrialists should not only strengthen their management level, but also enhance their innovation ability. Jalili et al. (2013^12^) used DEA to study the efficiency of 28 pharmaceutical manufacturing enterprises to measure and identify productivity changes in some pharmaceutical companies^12^. Mahajan et al. used DEA to estimate the technical efficiency of large pharmaceutical companies in India^13^. Mao et al. evaluated the operating efficiency of 34 Chinese pharmaceutical companies by using DEA^14^. They considered three inputs (labor force size, overhead, and total assets) and one output (operating income). The study concluded that the overall efficiency of the sector in China is not high. Based on the two-stage DEA method, Shimura et al. used one input (R&D expenditures) and three outputs (sales, operating profit, and the cumulative number of weighted new molecular entities approved by the Japanese Ministry of Health and Welfare) to measure the R&D productivity of pharmaceutical manufacturing enterprises^15^. Alam and Rastogi used the DEA method to evaluate the efficiency of five pharmaceutical industries in India^16^. Gasco´n et al. used the DEA method to evaluate the efficiency of 37 large pharmaceutical laboratories in the United States^17^. Mahajan et al. studied the efficiency of factor pharmaceutical enterprises and explored its determinants^18^. Díaz & Sanchez measured the efficiency performance of European pharmaceutical enterprises by DEA method, and found that TFP showed a downward trend^19^. Liu & Lyu evaluated the TFP of the pharmaceutical industry in China using the dynamic DEA method. It is found that its TFP is low due to the low efficiency of pure technology and scale, among which the main reason is the low efficiency of pure technology^20^. Zhong et al. used the super network SBM model and the Global-Malmquist (GM) index to study the innovation TFP of the pharmaceutical manufacturing industry^21^. Guan et al. used DEA-Malmquist index to evaluate the efficiency of China's pharmaceutical manufacturing industry from three stages (innovation production process, innovation integration process and innovation application process)^22^. The results showed that the overall efficiency of China's pharmaceutical manufacturing industry fluctuates.

It is summarized that there is no lack of academic research on measuring TFP in the pharmaceutical industry based on the macro perspective, as well as there are studies on measuring the efficiency of pharmaceutical enterprises in western countries or regions, but there seems to be a lack of research on the efficiency of Chinese pharmaceutical enterprises.

### Corporate governance structure and TFP

When reviewing the literature, we did not find any literature analyzing the relationship between governance structure and TFP of pharmaceutical enterprises. Because the research object of this project is enterprise, this section studies the existing literature on the relationship between corporate governance structure and enterprises’ TFP, aiming to provide some literature support for this work.

Tian & Twite (2011) took the sample of Australian companies as an example to investigate the influence of internal corporate governance on TFP^23^. The results showed that internal corporate governance mechanism can significantly affect corporate productivity. Su and He studied the impact of corporate governance on production efficiency based on a sample of 744 listed manufacturing enterprises in China from 1999 to 2006^24^. It was found that improving corporate governance structure can improve the efficiency of enterprises. Albulescu and Turcu studied the impact of corporate governance structure on the TFP of Romanian R&D firms^25^. It is found that the degree of independence of decision making and the presence of owners in business management have a negative impact on TFP. Min & Smyth took Korean enterprises as an example and found that the improvement of corporate governance structure had a positive impact on the TFP of enterprises^26^. Gaitán et al. (2018) studied the relationship between corporate governance and productivity of non-financial listed companies in Latin America, and found that board size, gender diversity, institutional ownership and the presence of independent directors affected the productivity of companies^27^. Andries et al. studied the impact of corporate governance on TFP based on 139 commercial banks from 17 countries in Central and Eastern Europe. The results show that the level of efficiency is related to corporate governance structure. He et al. found that corporate governance had a significant impact on the manufacturing efficiency and the efficiency of various input factors^28^. Nguyen & Vo (2020) used the dynamic system GMM model to assess the impact of corporate governance on the banks’ TFP measured by the SFA model. It was found that the nature of banks and board size had an impact on the TFP, while foreign ownership, board independence and CEO duality have no significant impact on the TFP^29^. Kong & Kong found that corporate governance had a significant impact on the TFP of Chinese enterprises, and corporate governance also had moderating and mediating effects between human capital and TFP^30^. Improving corporate governance can greatly improve TFP. Shabbir et al. studied the impact of corporate governance on efficiency (measured by TFP calculated by DEA) of Chinese Internet companies^31^.

In summary, we found that there were many papers researched the impact of corporate governance on TFP in academia. But there is a lack of research on pharmaceutical enterprises. This is a work in urgent need of supplement.

The possible innovations of this study are shown below. First, the innovation of research perspective. To our knowledge, no study researched the link between corporate governance and TFP in pharmaceutical firms. This article is the first time to study the impact configurations of corporate governance structure on TFP of pharmaceutical enterprises from the perspective of internal medicine enterprises. It fills the research blank in this area. Second, the innovation of research methods. In the process of measuring the TFP of pharmaceutical enterprises, this study combines the Super SBM model and the GM index model. The efficiency of pharmaceutical enterprises can be estimated more accurately. In addition, in the process of analyzing the influencing factors, this paper uses the Qualitative Comparative Analysis of Fuzzy Sets (fsQCA) method. This method is especially helpful to analyze small and medium-sized samples and avoid only considering dichotomous variables.

## Research hypothesis

The factors that affect enterprise TFP include internal factors and external factors. The external factors are caused by social and market reasons, and enterprises cannot control the external factors. Internal factors are caused by the enterprise itself, and the enterprise can control itself. Because this paper locates the angle of view in the enterprise itself. Therefore, we carry on the consideration of the enterprise’s internal factors. According to Cadbury (1992), corporate governance brings order to the organization. Corporate governance focuses on monitoring and control structures to incentivize and motivate managers, minimize agency problems, and protect the rights of shareholders^31^. In China, due to the development and listing of more and more companies, corporate governance has been increasingly valued.

The governance structure of a company is mainly three meetings and one layer, i.e., the shareholders' meeting, the board of directors, the supervisory board and the senior management. This paper mainly selects the variables referring to corporate governance from the aspects of shareholders, directors and supervisors. In terms of shareholders, this paper selects equity concentration; in terms of directors, this paper selects board size, proportion of independent directors and board activities; in terms of supervisors, this paper selects supervisory board size. Agency theory suggests that there is a principal-agent relationship within a pharmaceutical company, i.e., the principal entrusts the agent to manage and operate the company. On the basis of agency theory, corporate governance affects TFP of pharmaceutical enterprises through two paths. First, agency theory suggests that agents may have problems prioritizing their own interests over those of the principal. By establishing effective monitoring and control mechanisms, corporate governance can ensure that agents act in the interests of principals and motivate agents to fulfill their duties. Therefore, the establishment of independent directors and supervisory boards, for example, can provide an effective monitoring mechanism to prevent agents from abusing their power or violating the interests of the principal. Second, the TFP of pharmaceutical companies depends on high-quality decision-making, and high-quality decision-making needs to be based on accurate and timely information. However, as the number of board members increases, the effectiveness and timeliness of information communication decrease, and information asymmetry intensifies, further affecting the company's decision-making ability^32,33^. Providing comprehensive and timely information disclosure and establishing effective communication mechanisms can reduce information asymmetry, and improve the accuracy and efficiency of decision-making ^34,35^.

In order to measure the impact of corporate governance on the TFP of pharmaceutical enterprises, this study focuses on five important aspects of corporate governance: ownership concentration, board size, proportion of independent directors, board activity and supervisory board scale. Agency theory is often used to explain corporate governance, so this paper mainly applies agency theory in the research hypothesis.

### Ownership concentration

A large number of studies have confirmed that there is a significant relationship between ownership concentration and enterprise efficiency. Ownership concentration can enhance the ability and behavior of large shareholders to supervise and restrain corporate accounting, and restrict agency costs^36,37^. So that the enterprise management can enable corporate management to make continuous efforts to realize corporate value and shareholder interests. In addition, ownership concentration is closely related to corporate financing, operation and management decisions. As high-tech enterprises, pharmaceutical enterprises have higher requirements for development decision-making, product research and development, marketing and customer service. The higher the ownership concentration is, the more beneficial it is for the management to make the right decisions, and the TFP of the enterprise will also be improved. This paper predicts that companies with high concentration have higher TFP efficiency. Therefore, this study proposes Hypothesis 1.

H1: Ownership concentration can improve the TFP of pharmaceutical enterprises.

### Board size

The board size plays an important role in the corporate structure. On the one hand, the board size, as the agent of shareholders, is the decision-making body of the daily affairs of enterprises. On the other hand, the board of directors is also the principal of the management. Therefore, the board size is likely to have an impact on the efficiency of enterprises. In economic theory, the scale effect is inverted U-shaped. Studies have shown that the larger the board size is, the more serious the agency problem^38,39^. In other words, there is a negative correlation between enterprise efficiency and board size^40^. Large boards have problems communicating with other levels due to their sheer size^41,42^. In addition, strategic management is also an important direction. Large-scale boards limit the ability of members to initiate strategic interactions^43^. In contrast, small boards are more effective in communicating information and meeting the interests of stakeholders because of the flexibility and cohesiveness of their small size^31^. Other studies shown that the impact of board size on enterprise efficiency is also affected by ownership concentration^44^. In the case of ownership concentration, the board size will have a negative impact on TFP of enterprises. Therefore, Hypothesis 2 is proposed in this study.

H2: The board size has a negative impact on the TFP of pharmaceutical enterprises.

### Proportion of independent directors

Board independence means that a director has no relationship with the company other than as a director. The independence of the board of directors can ease the supervision and control of managers, thereby reducing potential agency problems^39,45^. On the other hand, in listed companies, independent directors can also effectively protect the rights and interests of minority shareholders. Once the insiders encroach on the interests of external minority shareholders, independent directors can effectively play their personal responsibilities to ensure that the interests of minority shareholders will not be damaged, thus indirectly promoting the efficiency of enterprises. Therefore, this study proposes Hypothesis 3.

H3: The proportion of independent directors has a positive impact on the TFP of pharmaceutical enterprises.

### Board activity

The frequency of board meetings reflects its diligence and activity. The board of directors makes decisions through meetings^46^. Increasing the number of meetings can improve management and maximize the value of the enterprise. But there is also another possibility that the frequent board meetings may be held to deal with the risks faced by enterprises. In this case, the convening of the board of directors can rectify the problems existing in the enterprise, which is also conducive to the follow-up development of the enterprise. Therefore, this paper puts forward hypothesis 4.

H4: The board activity has a positive effect on the TFP of pharmaceutical enterprises.

### Supervisory board scale

The board of supervisors is the standing supervisory body of the company under the leadership of the shareholders' meeting, which performs supervisory functions. In order to ensure the normal and orderly operation of the company, to ensure that the company's decision-making is correct and the senior management can perform their duties correctly, and to prevent abuse of power and endanger the interests of the company, shareholders and the third party, all countries stipulate that the board of supervisors should be set up in the company^26^. The board of supervisors can supervise and restrain the behavior of hidden actions and hidden information of management, which is helpful to enhance the value of enterprises. Because of the special functions of the board of supervisors, within a certain range, the larger the size of the board of supervisors, the higher the efficiency of pharmaceutical enterprises.

H5: The supervisory board scale has a positive impact on the TFP of pharmaceutical enterprises.

**Figure 2:**
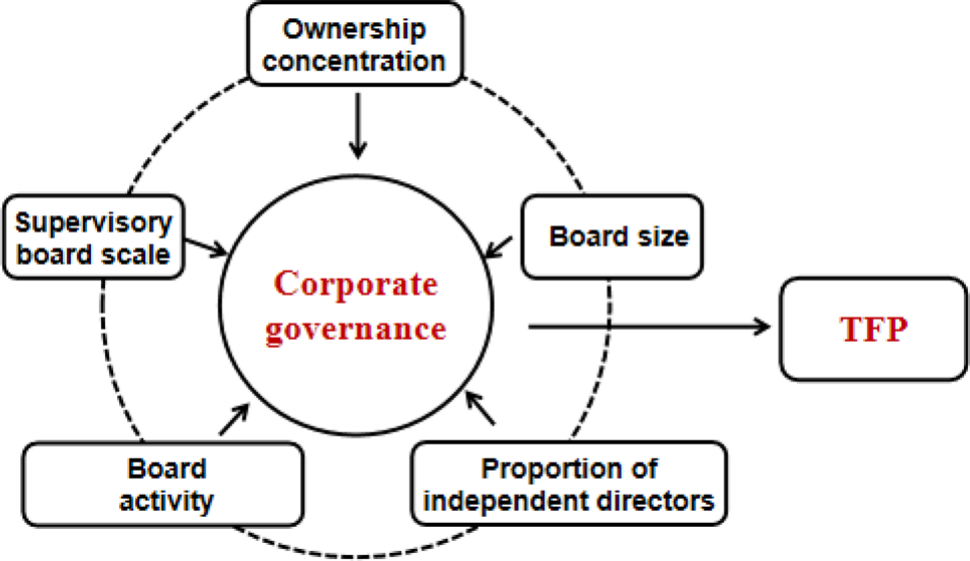
Theoretical model.

The theoretical model is shown in Fig. 2, and results of the five research hypotheses is shown in Table 1:

**Table 1:** Results of the five research hypotheses.

Variables	Research hypothesis
Equity concentration	+
Board size	−
Proportion of independent directors	+
Board activity	+
Supervisory board scale	+

## Measuring TFP of 47 listed pharmaceutical enterprises

### Research methods

In this paper, TFP is used to measure the comprehensive efficiency level of pharmaceutical enterprises, and the Super SBM-Global Malmquist index model is used to measure the TFP of the sample. Generally speaking, the methods of measuring efficiency mainly include SFA and DEA. SFA is a parametric method to estimate parameters such as cost or production function through maximum likelihood. DEA is a linear programming method for nonparametric estimation, which can handle problems with multiple inputs and outputs. Compared with SFA, DEA model has fewer restrictions and less subjective errors, so it is more widely used.

DEA method was first proposed by Farrell^47^. Although DEA model is suitable for evaluating the problem of multi-input and multi-output; however, there is the slack variable problem in the traditional DEA model. In order to solve this problem, Tone (2001) proposed SBM model based on slack measure, which directly adds slack variable to the objective function^48^. It can effectively make up for the defects of the traditional DEA model.

Subsequent scholars found a common phenomenon in most studies related to efficiency, that is, the efficiency state of multiple DMUs is 100%^49^, which is not conducive to further comparison between the optimal DMUs. The Super SBM takes the slack variable into account, and further deals with the efficiency of the optimal DMUs in the results, which is more suitable for comparing those DMUs that are all in the efficient frontier of production^50^, and is in line with the purpose of this study. In general, there are two reasons for choosing the super-efficient SBM-GMI model in this paper: first, compared with the SFA model, the SBM does not need to set a specific form of the production function, so the SBM model is more advantageous when dealing with complex and nonlinear production processes. Moreover, the SBM model can consider both the input surplus and the output deficiency to give a more comprehensive efficiency evaluation, while the SFA model usually focuses on only one side of the input or output; secondly, compared with the EBM, MinDW and other models in DEA, the SBM model is a non-radial measurement model, which can consider both the input surplus and the output deficiency to get a more comprehensive efficiency evaluation; thirdly, compared with the SFA model, the SBM model does not need to set a specific production function form, so it is more advantageous when dealing with complex and non-linear production processes. Efficiency evaluation; third, compared with the SBM model, the advantage of the super-efficient SBM chosen in this paper is that the model has stronger differentiation ability. In the case that all decision-making units (DMUs) are efficient (i.e., the efficiency score is 1), the traditional SBM model cannot further distinguish the efficiency level of DMUs. The super-efficient SBM model, on the other hand, can further differentiate the efficiency level of effective DMUs on this basis, and thus has a stronger differentiation ability. The specific expression of the Super SBM model is as follows:

$$\uprho =\mathrm{min\, \rho }\frac{1-\frac{1}{m}\sum_{i=1}^{m}\left(\frac{{s}_{i}^{-}}{{x}_{ik}}\right)}{1+ \frac{1}{q}\sum_{r=1}^{q}\left(\frac{{s}_{i}^{+}}{{y}_{rk}}\right)}$$

$${\text{s}}.\,\mathrm{ t}.\, {x}_{ik}={\sum }_{j=1, j\ne k}^{n}{x}_{ij}{\lambda }_{j}-{s}_{i}^{-}$$

$${y}_{rk}={\sum }_{j=1, j\ne k}^{n}{y}_{rj}{\lambda }_{j}-{s}_{i}^{-}$$

$$\lambda \ge 0,\, {{\text{s}}}^{-}\ge 0,\, {{\text{s}}}^{+}\ge 0$$

3.1 $${\text{i}}=\mathrm{1,2},...,{\text{m}};\mathrm{ r}=\mathrm{1,2},...,{\text{q}};\mathrm{ j}=\mathrm{1,2},...,{\text{n}}({\text{j}}\ne {\text{k}})$$

where m stands for input, Q stands for output, and ${\text{s}}=\left({{\text{s}}}_{{\text{i}}}^{-}, {{\text{s}}}_{{\text{i}}}^{+}\right)$ represents, the amount of slack in inputs (X_i_) and outputs (y_r_).$\lambda$ represents the weight vector.

This paper combines Super SBM-Global Malmquist index (GMI) theory to measure the TFP of pharmaceutical enterprises, that is, Super SBM-GMI model. The GMI is a Malmquist index calculation method proposed by Pastor and Lovell^51^. It takes the sum of each period as the reference set, that is, the common reference set of each period is:

3.2 $${P}^{G}\left(x\right)={P}^{1}\left({x}^{1}\right)\cup {P}^{2}\left({x}^{2}\right)\cup \dots \cup {P}^{T}\left({x}^{T}\right).$$

Because each period refers to the same frontier, the calculation results in a single Malmquist index. The TFP in the GMI can be defined as:

3.3 $${TFP}_{t}^{t+1}=\frac{{E}^{g}\left({x}^{t+1},{y}^{t+1}\right)}{{E}^{g}\left({x}^{t},{y}^{t}\right)}.$$

EC is calculated as follows:

3.4 $$EC=\frac{{E}^{t+1}\left({x}^{t+1},{y}^{t+1}\right)}{{E}^{t}\left({x}^{t},{y}^{t}\right)}.$$

The closeness of the front t + 1 to the global front can be determined by $\frac{{E}^{g}\left({x}^{t+1},{y}^{t+1}\right)}{{E}^{t+1}\left({x}^{t+1},{y}^{t+1}\right)}$. The larger the ratio, the closer the leading edge t + 1 is to the global leading edge; the closeness of the front t to the global front can be determined by $\frac{{E}^{g}\left({x}^{t},{y}^{t}\right)}{{E}^{t}\left({x}^{t},{y}^{t}\right)}$. The larger the ratio is, the closer the front t is to the global front; The variation of the leading edge t + 1 compared to the leading edge t can be represented by the ratio of two ratios:

3.5 $${TC}_{g}=\frac{\frac{{E}^{g}\left({x}^{t+1},{y}^{t+1}\right)}{{E}^{t+1}\left({x}^{t+1},{y}^{t+1}\right)}}{\frac{{E}^{g}\left({x}^{t},{y}^{t}\right)}{{E}^{t}\left({x}^{t},{y}^{t}\right)}}=\frac{{E}^{g}\left({x}^{t+1},{y}^{t+1}\right)}{{E}^{t+1}\left({x}^{t+1},{y}^{t+1}\right)}\frac{{E}^{t}\left({x}^{t},{y}^{t}\right)}{{E}^{g}\left({x}^{t},{y}^{t}\right)}.$$

According to Wang et al.^8^, MI can be decomposed into efficiency change (EC) and technology change (TC):

3.6 $${TFP}_{t}^{t+1}=\frac{{E}^{g}\left({x}^{t+1},{y}^{t+1}\right)}{{E}^{g}\left({x}^{t},{y}^{t}\right)}=\frac{{E}^{t+1}\left({x}^{t+1},{y}^{t+1}\right)}{{E}^{t}\left({x}^{t},{y}^{t}\right)}\left[\frac{{E}^{g}\left({x}^{t+1},{y}^{t+1}\right)}{{E}^{t+1}\left({x}^{t+1},{y}^{t+1}\right)}\frac{{E}^{t}\left({x}^{t},{y}^{t}\right)}{{E}^{g}\left({x}^{t},{y}^{t}\right)}\right]=EC*TC$$

Referring to the existing literature, this paper takes the number of R&D personnel as the labor input and the total R&D investment and operating cost as the capital input, which constitute the variables for calculating the TFP input. When choosing TFP output variables, this paper mainly considers that TFP inputs and outputs should be logically related, so it chooses invention patents, return on net assets and main business income as the expected outputs of TFP.

### Index selection

In this paper, Super SBM-GMI is used to measure the TFP of pharmaceutical enterprises, and the evaluation index system of TFP of pharmaceutical enterprises is constructed (Table 2). The input indicators are total R&D investment, the number of R&D personnel and operating costs, while the output indicators are return on net assets, invention patents and operating income. The meaning of the indicator is as follows:

1. Total R&D investment: R&D investment is the key factor to determine the success or failure of pharmaceutical enterprises. As a high-tech enterprise with high investment, the amount of R&D investment of pharmaceutical enterprises greatly affects the success of drug R&D^52^. (When selecting pharmaceutical enterprises, this paper directly excludes the enterprises with less R&D investment which is not in line with the reality, and excludes the impact of such enterprises with strong sales ability which have achieved good economic benefits by illegal market operation on the result analysis.)
2. Number of R&D personnel: The number of R&D personnel represents the investment of human capital in the process of pharmaceutical research and development. As a high-tech enterprise, R&D personnel are the core of its innovation ability and the key factor for the success of drug research and development and whether it can bring huge profits to the enterprise^52^.
3. Operating cost: For pharmaceutical enterprises, operating cost refers to the cost of selling drugs or providing services to the outside world. Operating cost is selected as one of the indicators to measure its input^53^.
4. Return on equity: Return on equity is also known as return on shareholders' equity, return on net worth, etc. Return on equity can be used to measure the operating performance of listed pharmaceutical companies, which mainly refers to the profit income brought by shareholder investment^54^.
5. Main business income: This paper mainly evaluates the TFP of pharmaceutical enterprises, so the main business income is selected as one of the indicators to measure their output, which can avoid the impact of some non-drug sales business on the analysis results^55^.
6. Invention patents: Invention patents represent the innovation capability of pharmaceutical enterprises. The more the number of patent applications for invention, the stronger the innovation capability in this field, and vice versa^56^.

**Table 2:** Inputs and outputs for DEA model.

Index type	Index	Unit
Input	Total R&D investment	Ten thousand Yuan
	Number of R&D personnel	People
	Operating cost	Ten thousand Yuan
Output	Invention patent	Piece
	Return on equity	Percentage
	Main operating income	Ten thousand Yuan

### Measurement of TFP of 47 listed pharmaceutical companies in 2021

This paper uses the software MaxDEA 8.0 to analyze the TFP of pharmaceutical enterprises, and the results are shown in Table 3. From the table, we can see that the development of pharmaceutical enterprises is not balanced, and there are only 16 enterprises whose TFP is greater than 1. The EC and TC of Northeast Pharmaceutical (000597), Hainan Pharmaceutical (000566), Haizheng Pharmaceutical (600276) and Tianyao Co., Ltd (600488) are all greater than 1, which shows that the efficiency and technical efficiency of these four enterprises have improved, making them more competitive in the fierce competitive market, and then improving TFP. Among the 16 companies whose TFP is greater than 1, Huaren Pharmaceutical (300110), Kelun Pharmaceutical (002422), Qianhong Pharmaceutical (002550), Qianyuan Pharmaceutical (300254), Shapuace (603168), Teyi Pharmaceutical (002728), Yuheng Pharmaceutical (002437) and Zhejiang Pharmaceutical (600216) all have TC greater than 1, but EC is less than 1, indicating that these 8 pharmaceutical enterprises mainly improve their R&D capabilities in science and technology. Drive the growth of TFP and promote the development of enterprises. Anke Biology (300009), Taiji Group (600129), Xinlitai (000756) and Zhifei Biology (600216) are four companies with TFP greater than 1. Their EC is greater than 1, but TC is less than 1, which makes a major contribution to the improvement of efficiency. The enhancement of efficiency leads to an improvement in the company's TFP, and there is still potential for further advancement in the realm of technology research and development.

**Table 3:** TFP of 47 pharmaceutical companies in 2021.

DMU	TFP	EC	TC	DMU	TFP	EC	TC
300009	1.1988	1.8139	0.6609	300016	0.9869	0.9267	1.0650
300255	0.8659	0.8320	1.0407	000597	1.0198	1.0032	1.0165
002675	0.9187	0.8352	1.1000	002262	0.9665	1.0526	0.9182
300267	0.9595	1.1016	0.8709	000153	0.9749	1.0295	0.9470
300194	0.8054	0.7269	1.1081	600196	0.9452	0.9452	1.0000
600664	0.4970	0.5511	0.9017	000566	1.2423	1.1886	1.0452
002653	0.8368	0.8662	0.9660	002099	0.8837	0.7783	1.1354
600267	1.0943	1.0264	1.0661	300199	0.8886	0.6879	1.2918
600276	0.5645	0.6366	0.8868	600812	0.8194	0.7910	1.0359
600521	0.8314	0.8015	1.0374	002007	0.8266	0.7521	1.0990
300110	1.0202	0.9984	1.0218	600062	0.7941	0.7430	1.0688
002020	0.8721	0.8252	1.0568	002422	1.0298	0.9748	1.0565
300006	0.8351	0.7734	1.0798	002393	0.8505	0.7743	1.0984
000513	0.9681	0.9420	1.0277	600513	0.9190	0.8290	1.1086
600789	0.9266	0.8379	1.1059	000739	0.8285	0.8108	1.0218
002550	1.0070	0.9117	1.1046	300254	1.0259	0.9616	1.0669
600079	0.9435	0.8523	1.1070	603168	1.0407	0.9655	1.0779
002038	0.9698	1.0908	0.8891	600129	1.0010	1.0753	0.9310
002728	1.1075	0.9865	1.1227	600161	0.9409	0.8985	1.0472
600488	1.1890	1.0656	1.1159	300142	0.9505	1.1377	0.8355
002332	0.9685	0.8852	1.0941	600420	0.9552	0.8437	1.1322
000756	0.7565	0.7415	1.0202	002294	1.1929	1.2484	0.9555
002437	1.0240	0.8712	1.1754	600126	1.0078	0.9203	1.0951
300122	1.3659	1.6410	0.8324				

Among these 47 companies, there are 31 companies whose TFP is less than 1, and their market competitiveness is reduced. Further analysis of the reasons for the low TFP shows that among these 31 companies, there are 23 companies whose EC is less than 1 and TC is greater than 1, which shows that the improvement of technological level can not make up for the retrogression of efficiency and cannot obtain the benefits brought by technological progress, resulting in the reduction of TFP. The TFP of Enhua Pharmaceutical (002262), Erkang Pharmaceutical (300267), Fengyuan Pharmaceutical (000153), Shuanglu Pharmaceutical (002038) and Watson Biology (300142) is less than 1. Although EC is greater than 1, the technological research and development capabilities of these five companies have different levels of retrogression, indicating that the contribution of efficiency progress can not compensate for the impact of technological efficiency retrogression, thus affecting the competitiveness of these five companies in the market. Among the 31 low TFP enterprises, Harbin Pharmaceutical Co., Ltd. (600664), Haisco (002653) and Hengrui Pharmaceutical (600420), EC and TC are less than 1, efficiency changes and technological changes are at a retrogressive level, and the common retrogression of the two leads to the decline of TFP. If these three enterprises want to make steady progress in the fierce competition, they must improve their efficiency level and technology level. Bring the TFP to the optimal distance level.

Because different enterprises have different governance structures, which have different effects on TFP, TFP is taken as the result variable, and how to improve the governance structure to improve enterprise TFP is further discussed.

## Research methods and data

### fsQCA method

The basic idea of fsQCA method is to analyze the influence of multiple antecedent variables and outcome variables with the help of architecture theory and Boolean operations, so as to explain the complex causal mechanism behind the phenomenon^57,58^. The fsQCA method combines the "qualitative" and "quantitative" dimensions of a variable to some extent by assigning any value between 0 and 1 to the variable through a calibration procedure^59^. This paper uses fsQCA method to explore the nonlinear relationship between corporate governance and TFP of pharmaceutical enterprises, divides corporate governance into five factors (ownership concentration, board size, proportion of independent directors, board activity and board size), and excavates multiple equivalent configurations affecting TFP of pharmaceutical enterprises. It can also compare the asymmetric antecedents of high efficiency and non-high efficiency of pharmaceutical enterprises and deepen the research conclusions.

This paper was analyzed using the fsQCA4.0 software. Successive versions of QCA and fsQCA have been developed by Charles Ragin and Sean Davey. The software download address is given at https://sites.socsci.uci.edu/~cragin/fsQCA/software.shtml.

### Data sources and sample selection

This paper selects 47 listed pharmaceutical companies to analyze their data in 2021, which comes from the annual reports of listed companies and the China Stock Market & Accounting Research Database (CSMAR). There are 216 listed pharmaceutical companies in biomedicine and chemical medicine. Excluding traditional Chinese medicine enterprises, consulting services, veterinarians, diagnostic reagent enterprises, pharmaceutical distributors and enterprises whose main business is non-pharmaceutical R&D and production, and enterprises whose number of new drugs approved by the Food and Drug Administration for three consecutive years is zero. And this paper pluses the availability of other data. Finally, the remaining 47 eligible listed companies in the pharmaceutical industry were analyzed. (See Appendix 1 for the codes of 47 listed pharmaceutical companies). Sample selection principles: 1. Firms with missing data are excluded; 2. Firms with poor operating conditions, such as ST and ST*, are excluded.

This paper analyzes the data of 47 selected pharmaceutical listed companies in 2021, which comes from the annual reports of listed companies and the China Stock Market & Accounting Research Database (CSMAR). Specifically, the indicators measuring TFP and the five antecedent variables in this paper are derived from this database.

### Variable selection and calibration

#### Variable selection

##### Conditional variable

On the basis of previous studies on corporate governance, this paper comprehensively considers the number of cases and the number of factors in the fsQCA method and makes a reasonable design. This paper selects five variables as antecedents, namely, ownership concentration, board size, proportion of independent directors, number of board meetings and board size.

Ownership concentration (ONE). Ownership concentration is generally measured by the proportion of shares held by the top shareholders of the company. The distribution of shares will have different effects on the daily operation of the company and the decision-making of major issues. This paper uses "the proportion of shares held by the first shareholder" to express it.

Board Size (BOD). According to the principal-agent theory, the board of directors is mainly responsible for formulating strategies and plays an important role in key decisions. The size of the board of directors plays an important role in the development of the company, which is expressed by "the number of board members" in this paper.

Proportion of independent directors (ID). The proportion of independent directors refers to the ratio of the number of independent directors to the number of directors. The law stipulates that the number of independent directors on the board of directors shall be at least three, and shall not be less than one third of the total number of board members. The main responsibility of independent directors is to supervise the board of directors^60^, so that the management can manage the company under a more supervised and standardized management system.

Board Activity (Meetings). The number of meetings of the board of directors is the number of meetings held by the board of directors in the year, which is generally held to study major urgent matters, so the content and legal effect of the board is of great significance to the development of pharmaceutical enterprises. The more times the board meets during the year, the more active it is on behalf of the board, and vice versa.

Supervisory board scale (BOS). The board of supervisors is generally directly elected by the general meeting of shareholders, whose main responsibility is to exercise supervisory power on behalf of minority shareholders and assume responsibilities in the process of supervision, which is a very important factor in supervision, expressed by "the number of supervisors".

##### Result variable

In this paper, the TFP of pharmaceutical enterprises measured above is used as the result variable. TFP is used to evaluate the efficiency and productivity of decision-making units responsible for transforming inputs into production. When TFP > 1, the efficiency of pharmaceutical enterprises shows an upward trend. On the contrary, when TFP < 1, the efficiency of pharmaceutical enterprises decreases. Details of the variables can be found in the Table 4.

**Table 4:** Descriptive statistics of relevant variables.

Variable	Name	Obs	Mean	Std.dev	Min	Max
TFP	Total factor productivity	47	0.998	0.458	0.521	3.206
ONE	Ownership concentration	47	29.06	12.31	4.710	59.99
BOD	Board size	47	8.830	1.685	5	15
ID	Proportion of independent directors	47	0.366	0.0498	0.333	0.556
MEETINGS	Board activity	47	10.28	4.684	3	28
BOS	Supervisory board scale	47	18.26	4.748	10	35

#### Calibration

The relevant initial data were calibrated, and the three calibration points of full membership, intersection and full non-membership membership of the condition variable and the result variable were set to 0.9, 0.5 and 0.1 respectively. In this study, the data were calibrated on the basis of the existing results, and the complete membership points, cross membership points and complete non-membership points of the calibrated condition variables and result variables are shown in Table 5.

**Table 5:** Calibration of result and conditions.

Result and conditions	Anchors		
	Full membership	Maximum ambiguity	Full non membership
TFP	1.140112	0.955198	0.813821
ONE	47.998	26.6	16.57
BOD	11	9	7
ID	0.4286	0.3333	0.3333
MEETINGS	16	9	5.6
BOS	22.4	18	13

The specific names of the variables in the first column are shown in Table 4.

## Empirical analysis

### Necessary condition analysis

Before the condition configuration analysis, it is necessary to test the "necessity" of each condition separately. Consistency is an important test criterion for the necessary condition. When the consistency is greater than 0.9, the condition is the necessary condition for the result^61^. This paper uses fsQCA 3.0 software to test whether a single condition (including its non-set) constitutes a necessary condition for high TFP and low TFP in pharmaceutical enterprises. The results are shown in Table 4. It can be seen from Table 6 that the necessary consistency of a single condition variable to the high TFP or low TFP of pharmaceutical enterprises is less than 0.9, which does not constitute the necessary condition for the results, indicating that the explanation of a single corporate governance factor to the TFP of pharmaceutical enterprises is weak, and there is no necessary condition affecting the high TFP or low TFP.

**Table 6:** Necessity analysis of single conditions.

Condition	High TFP		Low TFP	
	Consistency	Coverage	Consistency	Coverage
ONE	0.65	0.64	0.70	0.71
~ ONE	0.71	0.70	0.65	0.65
BOD	0.65	0.68	0.63	0.67
~ BOD	0.68	0.64	0.70	0.67
ID	0.58	0.80	0.55	0.78
~ ID	0.84	0.64	0.86	0.68
MEETINGS	0.73	0.73	0.60	0.61
~ MEETINGS	0.61	0.60	0.74	0.74
BOS	0.64	0.70	0.61	0.67
~ BOS	0.70	0.63	0.73	0.68

The specific names of the variables in the first column are shown in Table 4.

### Sufficiency analysis of configuration

Different from the analysis of the above necessary conditions, configuration analysis mainly explores the adequacy of the results caused by different configurations composed of multiple conditions, and mainly studies which combinations of factors lead to the results. Based on the analysis of necessary conditions, this paper further analyzes the configuration of high TFP and non-high TFP in pharmaceutical enterprises. Referring to the previous studies, the original consistency threshold is set as 0.80^62^ and the Proportional Reduction in Inconsistency (PRI) threshold is set as 0.70 in the adequacy analysis of configuration. Since the number of cases is only 47, the case threshold is set to 1. After the relevant parameters are set, the standardization operation is carried out, and the core conditions and edge conditions in each configuration are obtained by comparing the complex solution, simple solution and intermediate solution. The final configuration results of high TFP and low TFP of pharmaceutical enterprises are shown in Table 7.

**Table 7:** Configurations strongly related to high TFP and low TFP.

Variables	High TFP			Low TFP
	High regulation		Active board	Negative board
Antecedent condition	H1a	H1b	H2	H3
ONE				
BOD				
ID				
MEETINGS				
BOS				
Consistency	0.8210	0.8513	0.8678	0.8504
Raw coverage	0.4237	0.4737	0.3763	0.5231
Unique coverage	0.0047	0.0272	0.1237	0.5231
Solution consistency	0.8136			0.8504
Solution coverage	0.6056			0.5231

Core casual condition (present).

Peripheral casual condition (present).

Core casual condition (absent).

Peripheral casual condition (absent).

Blank spaces indicate “do not care”.

The specific names of the variables in the first column are shown in Table 4.

1. The configuration of high TFP in pharmaceutical enterprises is as follows:
  According to Table 5, it can be concluded that there are three configurations that produce high TFP in pharmaceutical enterprises, and the consistency of both single solution and overall solution is greater than the acceptable standard of 0.75. Among them, the consistency of overall solution is 0.8136. The overall coverage is 0.6056, which is greater than the minimum standard 0.5. In these configurations, H1 is the regulatory constraint type, and H2 is the active board type. The specific analysis of each configuration is as follows.
  Configuration H1a: ONE* ~ ID*MEETINGS*BOS, that is “high ownership concentration* low proportion of independent directors *active board * high supervisory board scale”. This configuration can be interpreted as a regulatory constraint type. The configuration shows that the high supervisory board scale is the core condition, and the high ownership concentration, and the active board of directors are the marginal conditions. Under this configuration, the low proportion of independent directors will not affect the TFP development of pharmaceutical enterprises, and can achieve a high level of development of pharmaceutical enterprises with proper supervision. When the members of the board of supervisors perform their duties, they should make better supervision on the management of pharmaceutical enterprises from the perspective of the company's interests. At the same time, the hierarchical structure of pharmaceutical enterprises is relatively simple. The higher the concentration of ownership, the more conducive it is for the management to make correct decisions and promote the improvement of TFP of enterprises.
  Configuration H1b: BOD * ~ ID * MEETINGS * BOS, that is, high board size * low proportion of independent directors * board activity * high supervisory board scale. The configuration may also be of the regulatory constraint type. The size of the board of supervisors is the core condition, while the size and activity of the board of directors are the marginal conditions. Under this configuration, the lack of independent directors has no significant impact on the high TFP of pharmaceutical enterprises, while ensuring strong supervision, expanding the size of the board and improving the activity of the board of directors have a pulling effect on improving the TFP of enterprises.
  Configuration H2: ~ ONE * ~ BOD * ~ ID * MEETINGS * ~ BOS, that is, low ownership concentration * low board size * low proportion of independent directors * board activity * low supervisory board scale. This kind of configuration can be interpreted as the active board type. The number of board meetings is the core condition, and low ownership concentration, low board size, low proportion of independent directors and lack of supervision by the supervisory board are the marginal conditions. Under this configuration, we can see that the board meeting can be discussed by board members with different professional backgrounds, avoid the cognitive gap in decision-making, and make the collective decision-making more professional and scientific. At the same time, appropriately reducing the size of the board of directors, the proportion of independent directors and the supervisory board scale can indirectly improve the TFP of pharmaceutical enterprises. Due to the short rise time and rapid development of some pharmaceutical enterprises, the concentration of ownership is a double-edged sword for such emerging enterprises. In the short term, when the rights and interests of major shareholders conflict with the development of the company, they need to sacrifice the rights and interests of major shareholders in the short term to promote the development of the company. At the same time, the redundancy of board size will indirectly reduce the TFP of enterprises.
2. One configuration of low TFP for pharmaceutical enterprises is as follows:
  Configuration 3: ~ BOD * ~ ID * ~ MEETINGS * ~ BOS, that is, * low board size * low proportion of independent directors * low board activity * low supervisory board scale. This kind of configuration can be interpreted as the negative type of the board. The activity of the board of directors is the core missing condition, and the low board size, low board of supervisors size and low proportion of independent directors are the marginal conditions. In H3, ignoring the importance of the board meeting and the inactivity of the board of directors are the important factors that lead to the non-high TFP of pharmaceutical enterprises, while the small board size and the insufficient proportion of independent directors may also lead to the low TFP of pharmaceutical enterprises, which hinders the development of the company. The board of directors held fewer meetings in the year, and did not hold meetings according to the major or unexpected events that occurred in the year, resulting in the board of directors not formulating correct strategies and pointing out the clear direction of the company's development, resulting in damage to the company's interests, thus reducing the TFP of the enterprise. Especially for pharmaceutical enterprises, pharmaceutical enterprises are at the forefront of innovation, which will bring great profits to enterprises. They need to pay close attention to the latest developments in related fields in the world all the time. For related major events and emergencies, the board of directors needs to hold timely board meetings to formulate strategies in line with the company's highest interests to ensure that the company is at the forefront of innovation and development. At the same time, the small size of the board of supervisors and the board of directors also leads to the lack of reasonable supervision of the company, which fails to effectively prevent operators from sacrificing the interests of the company for their own interests, resulting in damage to the interests of the company, thus reducing the TFP of enterprises.

### Robustness test

In this paper, the robustness of the high TFP configuration in pharmaceutical enterprises is tested. QCA is a set theory method. It is considered robust when slight changes in operations, such as changing the calibration point of data, adjusting the frequency of cases, and increasing the consistency threshold, produce a subset relationship between the results and do not change the substantive interpretation of the findings. In this paper, the consistency threshold is reset from 0.8 to 0.85, and the calibration process of the condition variable is changed. The three calibration points of complete membership, intersection and incomplete membership of the condition variable and the result variable are changed to 0.8, 0.5 and 0.2 respectively. The results are shown in Table 8. The test presents results in overall agreement with Table 5.

**Table 8:** Robustness test.

Variables	High TFP			Low TFP
	High regulation		Active board	Negative board
Antecedent condition	H1a	H1b	H2	H3
ONE				
BOD				
ID				
MEETINGS				
BOS				
Consistency	0.8156	0.8568	0.8534	0.8364
Raw coverage	0.3285	0.5216	0.4287	0.5254
Unique coverage	0.0087	0.0102	0.1468	0.5253
Solution consistency	0.8366			0.8513
Solution coverage	0.6245			0.5341

Core casual condition (present).

Peripheral casual condition (present).

Core casual condition (absent).

Peripheral casual condition (absent).

Blank spaces indicate “do not care”.

The specific names of the variables in the first column are shown in Table 4.

### Optimal configuration analysis

Referring to the existing literature, this paper defines the optimal grouping as the grouping with the highest original coverage. In order to effectively explore the dynamic relationship between corporate governance and TFP of pharmaceutical companies, this paper separately conducted a comparative grouping analysis for each of the years 2018–2022 and selected the optimal grouping, and the results can be seen in the table. From Table 9, it can be seen that the optimal grouping in 2021 is more similar to the grouping in the nearby years, which indicates that the conclusions of this paper can also be valid in other years.

**Table 9:** Optimal Configuration Table.

Variables	2018	2019	2020	2021	2022
ONE					
BOD					
ID					
MEETINGS					
BOS					

Core casual condition (present).

Peripheral casual condition (present).

Peripheral casual condition (absent).

Blank spaces indicate “do not care”.

The specific names of the variables in the first column are shown in Table 4.

### Discussion

This study focuses on the relationship between governance and total factor productivity of 47 listed pharmaceutical manufacturing companies in China. In terms of methodology, the combination of DEA model and panel data fsQCA is of great significance in clarifying the impact of enterprise led internal governance on improving total factor productivity.

Firstly, in terms of efficiency of Chinese pharmaceutical companies, research results show that in 2021, only a small number of Chinese pharmaceutical companies, especially 16 out of 47 pharmaceutical companies, had TFP greater than 1. Overall, the efficiency of China's pharmaceutical industry is relatively low, which is similar to previous research findings^63^. In addition, there are studies indicating that the innovation efficiency of the Chinese pharmaceutical industry is relatively low^21,64^. All of these indicate that there is great room for improvement in the efficiency of the industry.

Secondly, in terms of configuration analysis, this paper focuses on the high TFP configuration of pharmaceutical enterprises. The configuration H1a and configuration H1b have the same core conditions, both with high supervisory board size, only differing in terms of equity concentration and board size. This can be explained as a regulatory constrained governance structure. The typical enterprise that belongs to configuration H1 corporate governance structure is Northeast Pharmaceutical (000597), taking it as an example, according to the company's annual report, the board of supervisors of Northeast Pharmaceutical enterprises has a high scale, the board of directors has a high activity, the ownership concentration and the board scale are all above the middle level, but the proportion of independent directors of this enterprise is not high, which is barely in the middle level. The TFP of Northeast Pharmaceutical is 1.020, which is greater than 1 and is in an effective state. Northeast Pharmaceutical will realize "mixed reform" in 2019 and build a modern corporate governance system. Through this "mixed ownership reform", Northeast pharmaceutical enterprises have formed a new ownership structure, and constructed a new management mode with strict constraints, strong incentives and emphasis on implementation through the supervisory board, the concentration of ownership and the active board of directors, which promotes the improvement of TFP of enterprises and conforms to the typical characteristics of the concentration of ownership under the supervision of this paper.

As an active board configuration, typical companies with such corporate governance structures in configuration H2 include Shapu Aisi (603168). According to the company's annual report, Sharp's ownership concentration level is at the end of the sample pharmaceutical enterprises, the size of the board of directors is lower, the proportion of independent directors is at a medium level, and the size of the board of supervisors is also at a medium level, but its board of directors is more active. According to the TFP calculation, the TFP value of Sharp's is 1.041, which is in the effective state. Sharp's is listed as a national high-tech enterprise in China, with the characteristics of high investment, high innovation and high growth. Therefore, the board meeting of the company is held more frequently to meet the company's sustainable development in the high-tech field. With the continuous development of Sharp's enterprises, its ownership concentration, board size and the proportion of independent directors should also be adjusted accordingly to adapt to the changes in the size of enterprises.

By comparing configuration H1 and configuration H2, it is found that the main differences between H1 configuration enterprises and H2 configuration enterprises are as follows. Configuration H1 enterprises have sufficient size of the board of supervisors and sufficient supervision and checks and balances, which can ensure the implementation of correct decisions through the supervision of the board of supervisors on the company's operation, while avoiding abuse of power and protecting the interests of the company, shareholders and third parties. Li & Zhang (2023) also affirmed the role of the supervisory board in their research, pointing out that implementing internal supervision by the supervisory board can help improve the investment efficiency of enterprises^65^. In H1 configuration enterprises, the hidden actions and hidden information of management will be more likely to be supervised and constrained, so the normal order of enterprise operation will be more likely to be guaranteed, and its efficiency will be improved. This kind of configuration is more suitable for large-scale pharmaceutical enterprises. The existence of the board of supervisors is equivalent to the establishment of an independent information channel, and can effectively play the role of supervision and checks and balances. The characteristic of configuration H2 enterprises is the high activity of the board of directors. The board of directors of these enterprises shows a strong degree of diligence and has a high frequency of board meetings within a reasonable range. Frequent board meetings can make more reasonable decisions about the company's production and operation, while reducing the risks that may arise in such configuration enterprises. Cristina et al. proposed a similar viewpoint in their study of the impact of board social activities on corporate performance, indicating that a certain number of board meetings can maximize corporate profits^46^. Combined with the structure of configuration H2 and the actual situation of pharmaceutical enterprises in China, it can be concluded that H2 configuration is more suitable for pharmaceutical enterprises with decentralized equity. In the actual operation of enterprises, financing easily leads to the decentralization of equity, which is basically concentrated in small and medium-sized pharmaceutical enterprises. Therefore, it can also be said that small pharmaceutical enterprises are more suitable for H2 configuration. The activity of the board of directors can achieve the purposes of indirect control, scientific decision-making and executive incentives. Moreover, for some companies, the active board of directors is also equivalent to a formalism, which not only guarantees the direct control of the company's major shareholders, but also reflects the feelings of minority shareholders.

Through configuration analysis, we found that combining and synergizing different elements of corporate governance can effectively enhance the TFP of pharmaceutical companies. Enterprises should actively restructure their governance structure, appropriately increase the size of the supervisory board based on the company's size, strengthen the participation of the board of directors, and improve overall efficiency.

## Research conclusions and policy recommendations

### Research conclusion

**Figure 3:**
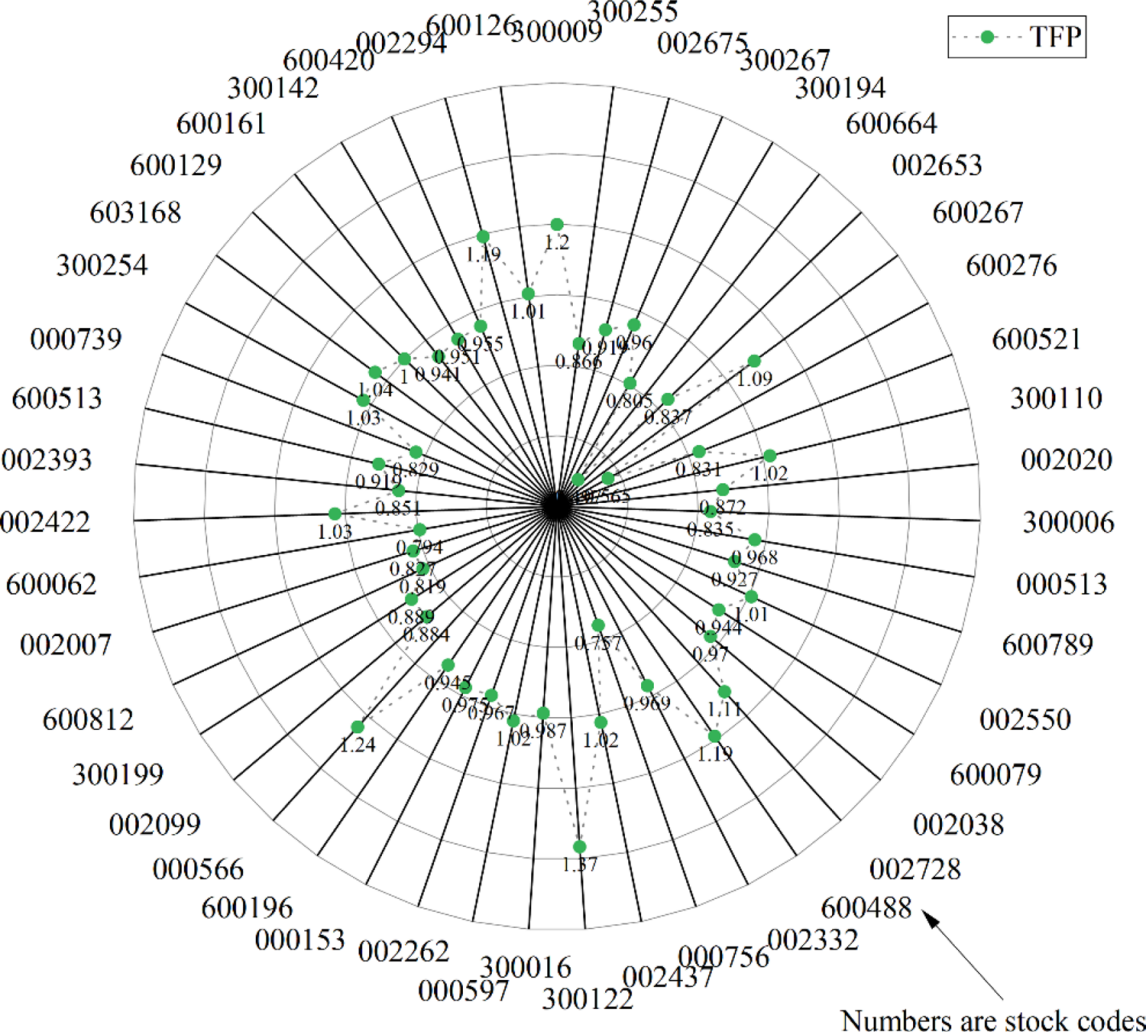
TFP of 47 pharmaceutical companies.

This paper first uses the Super SBM-Global Malmquist index model to measure the TFP of 47 pharmaceutical enterprises in China in 2021, and takes the TFP as the result variable. Based on the model of corporate governance factors, this paper uses the method of fsQCA to analyze the synergistic effects of five factors on the TFP of pharmaceutical enterprises, including ownership concentration, board size, the proportion of independent directors, board activity and the supervisory board scale. The main conclusions are as follows:

1. In 2021, among the 47 pharmaceutical enterprises in China, there are only 16 enterprises have TFP greater than 1, accounting for a relatively small proportion and unbalanced development, and pharmaceutical enterprises still have a large space for development, details can be found in Fig. 3.
2. Ownership concentration, board size, proportion of independent directors, board activity and supervisors cannot constitute the necessary conditions for high or low TFP of pharmaceutical enterprises. This indicates that a single condition has a weak impact on the TFP of pharmaceutical enterprises. Research has confirmed the complexity of corporate governance factors, and that it is possible to improve the TFP of pharmaceutical enterprises only if various factors are combined and linked.
3. There are three driving configurations for the formation of high TFP in pharmaceutical enterprises: two of them belong to the regulatory constraint type, and one belongs to the active board type. In different ways, reasonable increase the scale of supervision, improve the supervision and restraint of management behavior, to a certain extent, can prevent the management from doing harm to the interests of the company for their own sake. At the same time, improving the activity of the board of directors, holding board meetings in time for the risks and potential crises, rectifying the existing problems of enterprises and formulating relevant strategies will help to improve the TFP of pharmaceutical enterprises, enhance the competitiveness of pharmaceutical enterprises, and help enterprises to develop better in the future.
4. There is only one driving configuration of low TFP in pharmaceutical enterprises: the negative board type. In this configuration, the number of board meetings held in the year is too small. At the same time, the board of directors and the number of independent directors is too small, which leads to the inactive board of directors. It indicates that when the enterprise is facing danger or major decisions, the directors do not hold meetings in time, and there is no in-depth and effective discussion based on the actual situation. Due to the lack of effective discussion and the formulation of correct decisions, resulting in the reduction of supervision efficiency, there is no effective prevention of the company's losses caused by the management's self-serving behavior. The problems existing in the development have not been effectively solved, which will affect the development of pharmaceutical enterprises to a certain extent and reduce their competitiveness in the market.

### Policy recommendations

The research conclusion of this paper can provide the following two aspects of pharmaceutical corporate governance policy recommendations:

1. Expand the supervisory board scale, and improve the corresponding supervision system. It is necessary to reduce the harm caused by information asymmetry because managers make unfavorable actions for their own interests. In order to prevent the emergence of adverse situations, we can refer to some foreign enterprises. They will give their managers some equity incentives to link the interests of managers with the long-term interests of the company. In this way, supervisors are encouraged to work hard and make decisions that are beneficial to the interests of enterprises as far as possible. Therefore, in order to ensure the long-term and stable development of Chinese pharmaceutical enterprises in the future, listed companies can give managers a certain proportion of equity under the principle of retaining corporate control, so as to effectively promote the development of the company and ultimately achieve the goal of improving TFP.
2. Improve the board activity. Appropriately increasing the number of board meetings will help the board of directors to control the operating conditions of pharmaceutical enterprises. At the same time, At the same time, expand the number of board members with different professional backgrounds, avoid cognitive gaps in decision-making, and make collective decision-making more professional and scientific. Pharmaceutical companies have a short history of establishment, rapid development, and unique industry characteristics. The invention of a new technology may bring significant changes to the entire enterprise. It is even more necessary to discuss issues in development in a timely manner. Issues will be thoroughly discussed at meetings, where members of the board can better communicate and exchange information, which is conducive to the long-term development of the company.

### Study limitations

This paper uses the fsQCA method to study the combination and driving mode of corporate governance factors that affect the TFP of 47 pharmaceutical enterprises in China. The conclusions are helpful to understand the complex interaction among multiple factors to jointly promote the efficient development of pharmaceutical enterprises, and provide suggestions for improving the competitiveness of pharmaceutical enterprises. However, there are also the following limitations:

1. Although the TFP configuration analysis was conducted for 47 pharmaceutical enterprises, a specific case was not specifically analyzed. Subsequent research can be conducted based on panel data from specific enterprises to obtain a more targeted driving model for TFP of pharmaceutical enterprises.
2. Due to the limitations of the fsQCA method, cross-sectional data can only be used to analyze the TFP of pharmaceutical enterprises based on a static configuration perspective. As corporate governance factors become increasingly complex, the dynamic changes of various factors over time will be taken into account in the future to explore the dynamic impact of corporate governance on TFP of pharmaceutical enterprises.

### Supplementary Information

Supplementary Information.

## Data Availability

The datasets used and/or analysed during the current study available from the corresponding author on reasonable request.
