# Supplementary material for: The impact of corporate governance on the total factor productivity of pharmaceutical enterprises: a study based on the fsQCA method
Source: Sci Rep. 2024 Feb 8;14:3285. doi: 10.1038/s41598-024-52751-8 (PMC10853501; doi:10.1038/s41598-024-52751-8)
Supplement: Supplementary file 1 — Supplementary Information. [file 41598_2024_52751_MOESM1_ESM.docx]

**Appendix 1**

| **Company** | **Company code** | **Company** | **Company code** |
| --- | --- | --- | --- |
| Fengyuan Pharmaceutical | 000153 | Changshan Pharmaceutical | 300255 |
| Lizhu Group | 000513 | Erkang Pharmaceutical | 300267 |
| Hainan Haiyao | 000566 | China Resources Double-Crane | 600062 |
| Northeast Pharmaceutical | 000597 | Humanwell Medicine | 600079 |
| Pulo Pharmaceutical | 000739 | Taiji Group | 600129 |
| Xinhua Pharmaceutical | 000756 | Fosun Pharma | 600196 |
| Jingxin Pharmaceutical | 002020 | Zhejiang Medicine | 600216 |
| Shuanglu Pharmaceutical | 002038 | Haizheng Pharmaceutical | 600267 |
| Haixiang Pharmaceutical | 002099 | Hengrui Pharmaceutical | 600276 |
| Enhua Pharmaceutical | 002262 | Modern Chinese Medicine | 600420 |
| Xinlitai | 002294 | Tianyao Co., Ltd | 600488 |
| Xianju Pharmaceutical | 002332 | Lianhuan Pharmaceutical | 600513 |
| Lisheng Pharmaceutical | 002393 | Huahai Pharmaceutical | 600521 |
| Kelun Pharmaceutical | 002422 | Harbin Pharmaceutical Co., Ltd | 600664 |
| Yuheng Pharmaceutical | 002437 | North China Pharmaceutical | 600812 |
| Qianhong Pharmaceutical | 002550 | Shapuace | 603168 |
| Haisco | 002653 | Lukang Medicine | 600789 |
| Dongcheng Pharmaceutical | 002675 | Hualan Biology | 002007 |
| Teyi Pharmaceutical | 002728 | Tiantan Biology | 600161 |
| Laimei Pharmaceutical | 300006 | Anke Biology | 300009 |
| Beilu Pharmaceutical | 300016 | Zhifei Biology | 300122 |
| Huaren Pharmaceutical | 300110 | Watson Biology | 300142 |
| Fu'an Pharmaceutical | 300194 | Hanyu Pharmaceutical | 300199 |
| Qianyuan Pharmaceutical | 300254 |  |  |
